# Secondary hemophagocytic lymphohistiocytosis in pediatric patients: a single-center experience

**DOI:** 10.3389/fped.2026.1834746

**Published:** 2026-06-12

**Authors:** Mayada Abu Shanap, Aseel Bzour, Iyad Sultan, Rawad Rihani

**Affiliations:** Department of Pediatric Oncology, King Hussein Cancer Center, Amman, Jordan

**Keywords:** hyperferritinemia, infection-associated HLH, langerhans cell histiocytosis, macrophage activation syndrome, outcomes, pediatric, secondary HLH

## Abstract

**Background:**

Hemophagocytic lymphohistiocytosis (HLH) is a life-threatening hyperinflammatory syndrome. In children, secondary HLH may occur with infection, rheumatologic disease, malignancy, or metabolic disorders and is often difficult to diagnose because it overlaps with sepsis and other severe inflammatory states. We aimed to describe the etiologies, clinical features, treatment patterns, and outcomes of pediatric secondary HLH at a single tertiary center.

**Methods:**

We performed a retrospective review of children diagnosed with secondary HLH between April 2007 and June 2022. Diagnosis was based on HLH-2004 criteria. Demographic, clinical, laboratory, treatment, and outcome data were analyzed descriptively.

**Results:**

Sixteen children were included, with a median age at diagnosis of 34 months (range, 2.5–174); 62.5% were male. Infections were the most common trigger (56.3%), followed by rheumatologic disease/macrophage activation syndrome (18.8%), metabolic disorders (12.5%), malignancy (6.3%), and Langerhans cell histiocytosis (LCH) (6.3%). At presentation, fever and cytopenias affecting at least two lineages were present in 93.8%, splenomegaly in 87.5%, and hemophagocytosis in 81.3%. Hyperferritinemia was present in all patients. All patients received HLH-directed therapy together with treatment of the underlying trigger. Thirteen patients achieved complete remission, one had partial response, and two had refractory disease. Overall survival was 87.5%. Both deaths occurred in high-risk noninfectious HLH associated with Wolman disease and refractory LCH, whereas no deaths occurred in infection-associated HLH.

**Conclusion:**

Pediatric secondary HLH was most commonly infection-associated and had favorable outcomes when recognized early and treated promptly. In contrast, metabolic disease– and LCH-associated HLH were linked to refractory disease and mortality.

## Introduction

Hemophagocytic lymphohistiocytosis (HLH) is a severe hyperinflammatory syndrome caused by uncontrolled activation of cytotoxic T lymphocytes, natural killer (NK) cells, and macrophages, resulting in excessive cytokine release, tissue infiltration, and progressive multiorgan dysfunction. In children, HLH often presents with persistent fever, hepatosplenomegaly, cytopenias, coagulopathy, hepatitis, neurologic manifestations, and extreme hyperferritinemia. If not recognized promptly, the syndrome may rapidly evolve into shock, liver failure, central nervous system involvement, and death. Contemporary pediatric literature emphasizes that HLH should be viewed not as a single disease, but as a clinical syndrome shared by a broad range of genetic and acquired disorders (1–4).

Traditionally, HLH has been classified into primary (genetic) and secondary (acquired) forms. Primary HLH is associated with inherited defects in lymphocyte cytotoxicity pathways, whereas secondary HLH develops in association with infections, rheumatologic and autoinflammatory disorders, malignancies, immune dysregulation, or metabolic disease (2, 5). However, this distinction is increasingly recognized as an oversimplification. Many children who appear to have “secondary” HLH may harbor genetic susceptibility or underlying immune defects that influence disease severity, recurrence risk, and treatment response (1). This evolving concept is particularly relevant in pediatric practice, where the threshold for genetic evaluation has become lower and the overlap between inherited and acquired inflammatory syndromes is better appreciated.

Despite growing awareness, diagnosis remains challenging. The HLH-2004 criteria are still the most widely used diagnostic framework and include molecular confirmation or fulfillment of at least five of eight criteria: fever, splenomegaly, cytopenias, hypertriglyceridemia and/or hypofibrinogenemia, hemophagocytosis, low or absent NK-cell activity, hyperferritinemia, and elevated soluble interleukin-2 receptor (sCD25) (6).

Yet in real-world pediatric settings, several of these tests are not rapidly available, especially NK-cell function and sCD25, and the syndrome frequently overlaps with sepsis, severe systemic inflammation, liver failure, or macrophage activation syndrome (MAS) (2, 7). Sen and colleagues highlighted that distinguishing HLH from systemic sepsis is one of the greatest diagnostic challenges in children and noted that ferritin values above 10,000 µg/L strongly support the diagnosis, although lower values do not exclude it (7). Accordingly, clinicians must rely on a combination of pattern recognition, serial laboratory assessment, and early search for an underlying trigger.

Among children with secondary HLH, infection-associated HLH is the most frequently encountered subtype. Viral infections are especially important, with Epstein–Barr virus (EBV) representing one of the best-recognized and most clinically significant triggers in pediatric HLH (8). EBV-driven HLH is particularly relevant because it may reflect either severe acquired immune activation or an underlying inborn error of immunity predisposing to defective control of EBV infection. In addition to infections, often termed MAS, is a well-established complication of systemic juvenile idiopathic arthritis and related inflammatory disorders. MAS shares major clinical and laboratory features with HLH, including fever, cytopenias, liver dysfunction, coagulopathy, and hyperferritinemia, but may emerge during apparent rheumatologic flare or infection and can be difficult to distinguish clinically (9, 10).

Malignancy-associated HLH is less common in children than in adults, but it carries substantial morbidity and mortality. Pediatric reports suggest that HLH may occur at malignancy presentations, during chemotherapy, or in association with infection complicating immunosuppression. Hematologic malignancies, especially lymphoid neoplasms and leukemia, predominate among reported pediatric cases, and outcomes are generally poorer than in infection-associated HLH (11, 12) Recognizing HLH in the oncology setting is especially difficult because many features overlap with treatment toxicity, marrow infiltration, and infection.

A smaller but increasingly important group of children develop HLH in association with rare diseases, including inborn errors of immunity and inborn errors of metabolism. Recent reviews have drawn attention to conditions such as lysinuric protein intolerance and lysosomal acid lipase deficiency (Wolman disease), which may present with fulminant HLH-like hyperinflammation (13) Likewise, metabolic reviews have documented HLH in association with disorders such as Gaucher disease, Niemann–Pick disease, propionic acidemia, methylmalonic acidemia, galactosialidosis, and COG6-related congenital disorders of glycosylation (14). These rare etiologies are highly relevant because failure to recognize the underlying diagnosis may delay disease-specific management and worsen outcome.

Given this broad etiologic spectrum, pediatric secondary HLH should be approached as a syndrome requiring urgent stabilization, rapid etiologic investigation, and tailored therapy directed both at the hyperinflammatory state and the precipitating disorder. Data from single-center pediatric cohorts remain valuable because they reflect real-world diagnostic pathways, treatment practices, and outcome patterns across heterogeneous triggers. The present study therefore aimed to characterize the underlying causes, clinical features, treatment strategies, and outcomes of pediatric secondary HLH in a single-center cohort, with particular attention to trigger distribution and factors associated with adverse outcome.

## Patients and methods

### Study design and setting

This was a retrospective, single-center cohort study conducted at a tertiary pediatric referral center. We reviewed the medical records of children younger than 18 years who were diagnosed with secondary hemophagocytic lymphohistiocytosis (HLH) between April 2007 and June 2022. Data were extracted from the medical records, the first manuscript draft, and the accompanying coded dataset. KHCC Institutional Review Board; study No. 17 KHCC 120.

### Case definition and eligibility

HLH was diagnosed according to the HLH-2004 diagnostic criteria, defined as fulfillment of at least 5 of 8 criteria. The evaluated criteria included fever, splenomegaly, cytopenias affecting at least two cell lineages, hypertriglyceridemia and/or hypofibrinogenemia, hemophagocytosis, low or absent NK-cell activity, hyperferritinemia, and elevated soluble interleukin-2 receptor (sCD25). Because NK-cell activity and sCD25 assays were unavailable at our center during the study period, diagnosis relied on the remaining HLH-2004 criteria in conjunction with multidisciplinary clinical assessment. Patients with known familial or primary HLH were excluded. Children were classified as having secondary HLH when an acquired or associated trigger was identified, including infection, rheumatologic disease/macrophage activation syndrome (MAS), malignancy, metabolic disease, or Langerhans cell histiocytosis (LCH). Given the recognized overlap between primary and secondary HLH, results of genetic testing for familial HLH and related immune dysregulation syndromes were reviewed when available.

### Data collection

The following data were collected for each patient: age at diagnosis, sex, underlying trigger or associated condition, clinical manifestations at presentation, laboratory findings, pathology findings, genetic testing results, treatment modalities, response to therapy, disease reactivation or relapse, need for hematopoietic stem cell transplantation (HSCT), and survival status at last follow-up.

Clinical variables included fever, splenomegaly, hepatomegaly when present, cytopenias, and hemophagocytosis identified on bone marrow or other tissue examination. Laboratory variables included ferritin, fibrinogen, fasting triglycerides, complete blood count parameters, bilirubin, liver enzymes, and coagulation parameters where available. HLH-2004 laboratory thresholds were used to classify findings whenever applicable.

### Treatment definitions

Treatment data included both HLH-directed therapy and trigger-directed therapy. HLH-directed therapy comprised corticosteroids, intravenous immunoglobulin (IVIG), cyclosporine, and/or etoposide-based regimens according to disease severity and clinician judgment. When used, dexamethasone was generally given at 10 mg/m^2^/day for 2–4 weeks during the initial control phase, etoposide at 150 mg/m^2^ per dose in selected severe or refractory cases, and cyclosporine at 6 mg/kg/day orally in two divided doses with dose adjustment according to renal function and therapeutic drug monitoring. Trigger-directed therapy included antimicrobial, antiviral, antifungal, rheumatologic, oncologic, metabolic, or other disease-specific treatments according to the identified underlying etiology.

### Outcome definitions

The primary outcome was overall survival (OS), defined as survival from HLH diagnosis to last follow-up or death from any cause. Secondary outcomes included treatment response, disease reactivation or relapse, and requirements for HSCT. Response to therapy was assessed clinically by resolution of fever, improvement in cytopenias and organomegaly, and decline in ferritin; Response to first-line therapy was categorized as follows: complete remission (CR) was defined as resolution of clinical HLH signs and normalization of laboratory diagnostic criteria; partial remission (PR) indicated improving but residual HLH activity with normalization of only part of laboratory diagnostic criteria, typically 3–5 of 6 criteria; refractory disease was defined as persistent or progressive HLH despite first-line therapy; and reactivation/relapse as recurrence of HLH after an initial response. For descriptive purposes, improvement occurring within several days to 2 weeks after treatment initiation was categorized as a rapid response, whereas improvement occurring after more than 2 weeks was categorized as a delayed response. Patients with persistent or progressive HLH despite therapy were considered to have poor or refractory response.

### Statistical analysis

Given the small sample size, analyses were primarily descriptive. Continuous variables are presented as median with range or median. Categorical variable therapy is summarized as frequencies and percentages. Because of the limited number of events, no formal multivariable analysis was performed, and associations with mortality were interpreted descriptively.

## Results

### Patient characteristics, clinical phenotype, and laboratory findings

Sixteen pediatric patients with secondary HLH were included as shown in (Table 1)*.* The median age at diagnosis was 34 months (range, 2.5–174), and 62.5% were male. All patients fulfilled at least 5 HLH-2004 criteria based on available data. At presentation, the cohort showed the characteristic features of HLH, including fever, cytopenias, splenomegaly, hemophagocytosis, and hyperferritinemia. Median hemoglobin was 8 g/dL (range, 6–9), median absolute neutrophil count was 300/µL (range, 100–450), median platelet count was 40 × 10^9/L (range, 20–80), and median ferritin was 6,217 ng/mL (range, 700–45,000). Additional abnormalities included elevated LDH in 6 patients, ALT >3× the upper limit of normal in 4, elevated CRP in all, INR ≥1.5 in 2, and bilirubin ≥2× the upper limit of normal in 2 patients. Hemodynamic instability at presentation was documented in 10 patients, and 9 required pediatric intensive care unit admission. Genetic testing was performed in 7 patients; one patient was found to carry a compound heterozygous SLC7A7 variant of uncertain significance, c.272C > A (p.Ala91Glu) and c.335G > A (p.Gly112Glu), consistent with lysinuric protein intolerance, and no pathogenic variants associated with classic familial HLH were identified in the remaining tested cases.

**Table 1:** Baseline characteristics, HLH phenotype, etiologies, treatment, and outcomes of pediatric secondary HLH (*N* = 16).

Domain/Variable	N (%)
Age at diagnosis, median (range)	34 months [2.5–174]
Male sex	10 (62.5%)
Genetic testing performed Positive	7 (43.8%) 1/7 (14%) (SLC7A7 gene)^a^
Etiology/trigger category Infection: EBV/CMV/COVID-19/Leishmaniasis/Tick Rheumatologic/MAS Metabolic: Lysinuric protein intolerance/Wolman disease Malignancy: AML LCH	9 (56.3%) 3 (18.8%) 2 (12.5%) 1 (6.3%) 1 (6.3%)
HLH features and laboratory markers. Fever Splenomegaly Cytopenias ≥2 lineages^b^ Hemophagocytosis (BM and/or LN) Ferritin, ng/mL, median (range) Ferritin ≥500 ng/mL Hypofibrinogenemia ≤150 mg/dL Hypertriglyceridemia ≥265 mg/dL	15 (93.8%) 14 (87.5%) 15 (93.8%) 13 (81.3%) 6,217 [700–45,000] 16 (100%) 10 (62.5%) 13 (81.3%)
HSCT performed	1 (6.3%)
Examples of trigger-directed therapy Infection-triggered HLH Malignancy-triggered HLH LCH-triggered HLH Rheumatologic/MAS Metabolic: -Wolman disease -Lysinuric protein intolerance	Dexamethasone/ IVIG; Ganciclovir, Rituximab, Amphotericin ± antibiotics Chemotherapy; HSCT LCH-directed therapy: prednisolone + vinblastine; escalated to dexamethasone + etoposide Dexamethasone Dexamethasone, Cyclosporine and Etoposide Dexamethasone + Disease-specific metabolic therapy
Status at last Follow-up Alive Dead Deaths in infection group Deaths in rheumatologic/MAS group Deaths in metabolic group Deaths in malignancy group Deaths in LCH group	14 (87.5%) 2 (12.5%) 0/9 (0) 0/3 (0) 1/2 (50%) 0/1 (0) 1/1 (100%)

This table summarizes the demographic characteristics, underlying triggers, clinical and laboratory features at diagnosis, selected treatment-related variables, and survival outcomes of children with secondary hemophagocytic lymphohistiocytosis treated at a single tertiary center. Continuous variables are presented as median and range, and categorical variables as *n* (%). Percentages were calculated using the number of evaluable patients for each variable when missing data were present. BM, bone marrow; CNS, central nervous system; HLH, hemophagocytic lymphohistiocytosis; HSCT, hematopoietic stem cell transplantation; IVIG, intravenous immunoglobulin; LCH, langerhans cell histiocytosis; LN, lymph node; MAS, macrophage activation syndrome.

a SLC7A7 recorded, consistent with lysinuric protein intolerance.

b Cytopenias (affecting ≥2 of 3 lineages in the peripheral blood): Hemoglobin <90 g/L, Platelets <100 × 109/L. Neutrophils <1.0 × 109/L.

### Etiologic distribution

**Figure 1:**
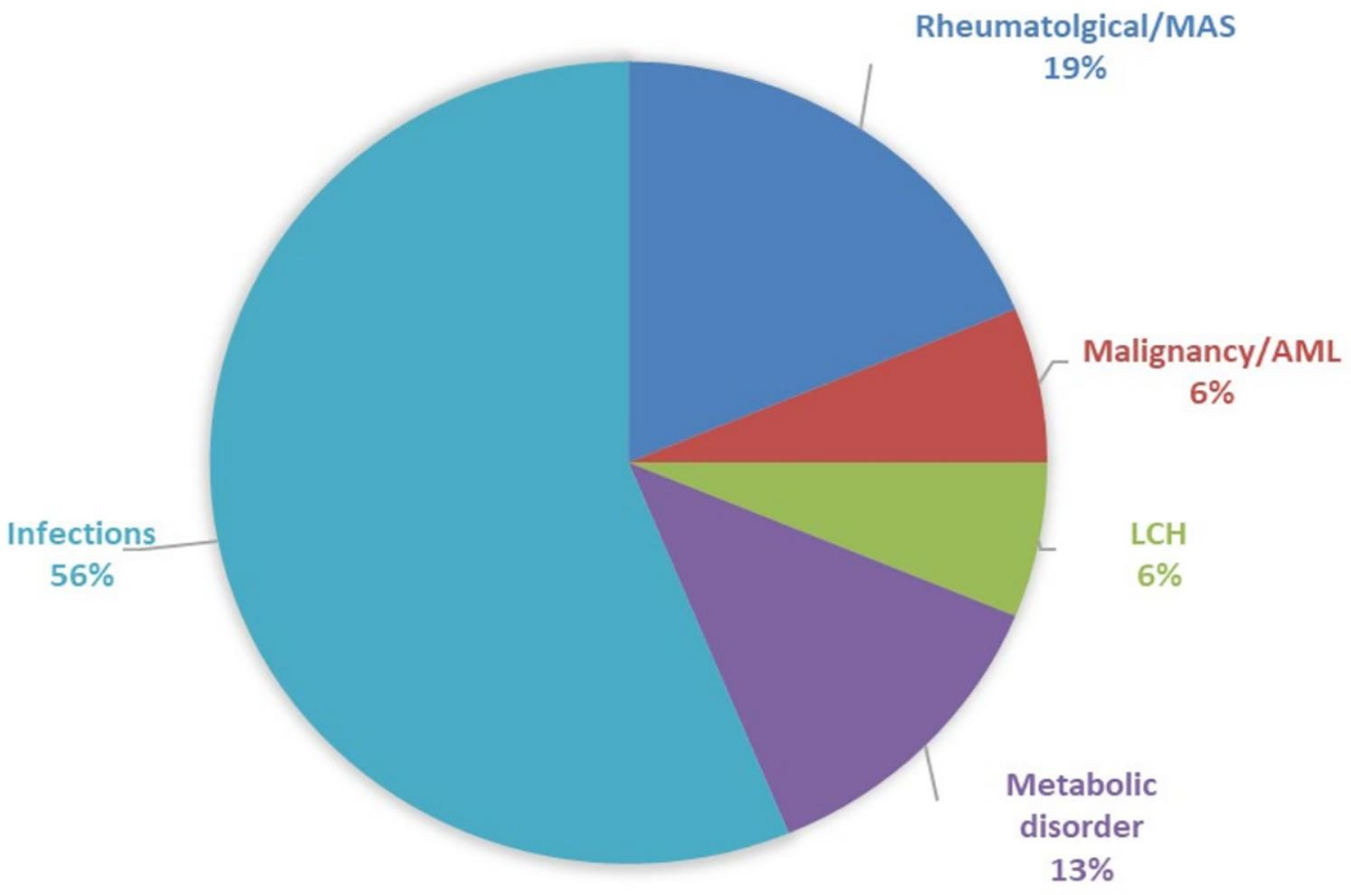
Etiologic distribution of secondary hemophagocytic lymphohistiocytosis in pediatric patients. Pie chart illustrating the distribution of underlying causes of secondary HLH in the study cohort, including infectious, rheumatologic/macrophage activation syndrome–associated, malignancy-associated, and other etiologies.

An underlying trigger was identified in all patients. Infections predominated, followed by rheumatologic/ Systemic juvenile idiopathic arthritis/macrophage activation syndrome (MAS), metabolic disorders, malignancy-associated HLH, and LCH (Figure 1). Infectious triggers included EBV, CMV, visceral leishmaniasis, tick-borne infection, and COVID-19, while the metabolic subgroup comprised lysinuric protein intolerance and Wolman disease (Table 1).

### Treatment patterns and outcomes

All patients received treatment directed at both the underlying trigger and the HLH-associated hyperinflammatory state. HLH-directed treatment consisted primarily of dexamethasone-based therapy, typically administered at 10 mg/m^2^/day for 2–4 weeks, with escalation to etoposide at 150 mg/m^2^ per dose and/or cyclosporine at 6 mg/kg/day orally in two divided doses in selected severe or refractory cases. Infection-associated cases were managed with targeted antimicrobial or antiviral therapy in combination with immunomodulation. Trigger-directed treatments included ganciclovir for CMV, rituximab for EBV-associated HLH, amphotericin with or without antimonial therapy for visceral leishmaniasis, antibiotics for tick-borne infection, and IVIG plus corticosteroids in COVID-19-associated cases.

The patient with malignancy-associated HLH had acute myeloid leukemia and was treated with cytarabine, daunorubicin, etoposide, and dexamethasone, followed by hematopoietic stem cell transplantation (HSCT) from a matched related donor. The patient with multisystem, risk-organ-positive Langerhans cell histiocytosis with secondary HLH received LCH-directed therapy as first-line treatment with prednisolone and vinblastine and, because of refractory disease, was escalated to dexamethasone and etoposide.

Patients with rheumatologic disease/macrophage activation syndrome (MAS) or metabolic disorders initially received empiric HLH-directed therapy, primarily dexamethasone, while primary HLH was being excluded, and were subsequently managed by the relevant specialty teams with disease-specific treatment. Patients with rheumatologic disease/MAS received disease-specific management, including IL-1 receptor antagonist therapy (anakinra). The patient with Wolman disease initially received empiric HLH-directed therapy with dexamethasone, followed by cyclosporine and etoposide. The patient with lysinuric protein intolerance received a short empiric course of dexamethasone and was subsequently managed with disease-specific metabolic therapy, including dietary protein restriction, oral citrulline supplementation, and L-lysine-HCl supplementation as indicated.

The overall survival of the cohort was 87.5% (14/16). Both deaths occurred in patients with high-risk non-infectious HLH: one in the metabolic subgroup (1/2, 50%) and one in the LCH-associated subgroup (1/1, 100%). In contrast, no deaths occurred among patients with infection-associated HLH or rheumatologic/MAS-associated HLH. The single patient with malignancy-associated HLH survived and successfully proceeded to HSCT. In clinically responsive patients, infection-associated HLH more commonly showed a rapid response, with improvement occurring within several days to 2 weeks after treatment initiation, whereas delayed response was more often observed in patients with noninfectious triggers. Poor or refractory response was seen in the highest risk cases, particularly Wolman disease and refractory multisystem LCH-associated HLH. At a median follow-up of 7 years (range, 2.8–18 years), no HLH reactivation or relapse was documented among survivors; late complications were not systematically captured.

## Discussion

In this retrospective single-center cohort of 16 pediatric patients with secondary HLH, we found that infection-associated HLH was the predominant clinical context, accounting for 56.3% of cases, followed by rheumatologic disease/macrophage activation syndrome (MAS) (18.8%), metabolic disorders (12.5%), malignancy (6.3%), and Langerhans cell histiocytosis (LCH) (6.3%). This distribution is broadly consistent with prior pediatric series, in which infections and rheumatologic/autoinflammatory disorders represent the most common triggers of secondary HLH, while malignancy-associated HLH is less frequent in children than in adults but usually carries a less favorable prognosis (8, 9, 12). Our findings therefore reinforce the concept that secondary HLH in children is a heterogeneous syndrome in which outcome is strongly influenced by the underlying trigger rather than by HLH itself as a single biological entity. Our findings further suggest that secondary HLH should not be viewed as a single prognostic entity. In our cohort, infection-associated HLH had the most favorable outcome, whereas rare noninfectious causes such as Wolman disease and refractory multisystem LCH were associated with poorer outcomes. This subgroup-based pattern emphasizes that, in pediatric secondary HLH, prognosis is driven largely by the nature, reversibility, and treatability of the underlying trigger.

The clinical phenotype in our cohort was typical for pediatric HLH. Nearly all patients had fever, splenomegaly, cytopenias affecting at least two cell lines, and marked hyperferritinemia, with a median ferritin of 6,217 ng/mL. Hypertriglyceridemia, hypofibrinogenemia, and hemophagocytosis were also frequent. These findings are consistent with the classic inflammatory profile described in pediatric HLH reviews and support current recommendations that HLH should be suspected in any child with persistent fever, cytopenias, organomegaly, and disproportionate hyperinflammation, particularly when the clinical course is more severe than expected for routine infection or inflammatory disease (2, 6, 7, 15).In real-world pediatric practice, diagnosis remains challenging because HLH frequently overlaps with sepsis, systemic inflammatory response syndromes, and MAS, and because some HLH-2004 investigations—particularly soluble IL-2 receptor and NK-cell function—may not be readily available (6, 10). In our cohort, all included patients fulfilled at least five HLH-2004 diagnostic criteria based on the available clinical, laboratory, and pathologic data. However, because NK-cell activity and soluble IL-2 receptor (sCD25) assays were unavailable at our center, diagnosis relied on the remaining HLH-2004 criteria together with multidisciplinary clinical assessment and exclusion of alternative explanations where relevant. This reflects a common real-world limitation in resource-constrained settings and should be considered when interpreting the diagnostic framework of this study.

An important observation in our series was the favorable overall survival of 87.5%, with no deaths among infection-associated cases. This outcome compares favorably with many previously published pediatric HLH cohorts and likely reflects the predominance of triggers that were identifiable and at least partly reversible with timely therapy. Prior studies have shown that infection-associated HLH often carries a better prognosis than malignancy-associated HLH, especially when the underlying infection is rapidly recognized and treated in parallel with immunomodulatory therapy (16, 17). In our cohort, infection-triggered cases included viral infections such as EBV, CMV, and COVID-19, parasitic infection such as visceral leishmaniasis, and other infectious/inflammatory presentations. This is clinically important because EBV remains one of the most important infectious triggers of pediatric HLH and may produce particularly severe immune activation, sometimes in the setting of an underlying predisposition to immune dysregulation (8). In such cases, adjunctive rituximab-containing therapy has been reported to reduce viral burden and improve inflammatory parameters when combined with HLH-directed therapy, which is consistent with the approach used in selected patients in our cohort (18, 19).

By contrast, the two deaths in our study occurred in children with high-risk non-infectious etiologies: one with Wolman disease and one with LCH-associated HLH. These observations are biologically plausible and consistent with the literature. Metabolic disorders are increasingly recognized as rare but important causes of HLH-like hyperinflammation in children, and several reports have highlighted lysosomal storage diseases, lysinuric protein intolerance, and other inborn errors of metabolism as conditions in which HLH may be severe, recurrent, or refractory to standard therapy unless the underlying disorder is specifically addressed (13, 14). Likewise, multisystem LCH can be complicated by a fulminant HLH-like inflammatory syndrome with high morbidity and mortality (20). Our experience supports the view that these rare etiologies deserve special consideration because they may not respond as favorably as infection-associated HLH to conventional immunosuppressive regimens alone. Although subgroup numbers were too small for formal comparative statistics, the cohort showed a clear qualitative pattern: reversible infectious and inflammatory triggers were generally associated with favorable response, whereas rare high-risk noninfectious conditions such as Wolman disease and refractory multisystem LCH were associated with treatment resistance and mortality. The rheumatologic/MAS subgroup also had favorable outcomes in our limited cohort, but its clinical course remained closely linked to control of the underlying inflammatory disorder.

From a treatment perspective, our cohort highlights the importance of a two-pronged strategy: rapid suppression of the hyperinflammatory process and simultaneous treatment of the underlying trigger. Most patients were managed with dexamethasone-based therapy, while a smaller subset required etoposide-containing regimens, generally reflecting greater clinical severity or higher-risk disease biology. This approach is consistent with contemporary HLH guidance, which emphasizes early immunomodulation once HLH is strongly suspected, followed by escalation to etoposide-based protocols in severe, refractory, or high-risk disease (21, 22). In MAS-associated HLH, evolving evidence also supports cytokine-directed approaches such as IL-1 blockade in selected patients, particularly in nonmalignant hyperinflammatory disease (9, 15, 23).

In the revised analysis, response was assessed clinically by defervescence, improvement in cytopenias and organomegaly, and decline in ferritin. Infection-associated HLH more commonly demonstrated a rapid response, generally within several days to 2 weeks after treatment initiation, whereas delayed or poor response was more often observed in patients with complex noninfectious triggers, particularly Wolman disease and refractory multisystem LCH-associated HLH. Because the study was retrospective and exact response timing was not uniformly documented for all patients, these response patterns should be interpreted qualitatively rather than as formal time-to-event comparisons. In addition, treatment was individualized according to etiology, disease severity, and clinician judgment over a long study period. While dexamethasone-based therapy was the most common initial HLH-directed approach, some patients also required cyclosporine, etoposide, or disease-specific therapies such as rituximab for EBV-associated HLH and anakinra in selected rheumatologic/MAS-associated cases. This therapeutic heterogeneity reflects real-world pediatric HLH practice but limits direct comparison of regimen-specific efficacy.

Although HLH has traditionally been divided into primary and secondary forms, this distinction is increasingly viewed as biologically overlapping rather than absolute. In general, primary HLH is associated with inherited defects in lymphocyte cytotoxicity, carries a higher risk of recurrence, and often requires etoposide-based therapy followed by allogeneic HSCT for definitive treatment. By contrast, secondary HLH is more often driven by an identifiable trigger such as infection, rheumatologic disease, malignancy, and outcomes may be favorable when both the hyperinflammatory state and the precipitating condition are treated early. Our cohort reflects this principle: infection-associated cases had favorable outcomes, whereas refractory disease and mortality were concentrated in patients with high-risk underlying disorders, underscoring that treatment intensity and prognosis in secondary HLH are heavily shaped by the trigger itself.

Another important finding in our study is the role of genetic and metabolic evaluation. Genetic testing was performed in 43.8% of patients, and although no classical familial HLH mutations were identified, one patient had lysinuric protein intolerance with an SLC7A7 variant, highlighting that some cases classified clinically as ’secondary HLH’ may instead represent HLH occurring in the setting of an inherited metabolic disorder or genetic predisposition rather than a purely acquired form. This finding reflects an increasingly recognized limitation of the traditional primary-vs.-secondary HLH classification. Pediatric HLH is now understood as a syndrome that may arise from a broad spectrum of underlying immune, genetic, rheumatologic, infectious, neoplastic, and metabolic disorders, with substantial overlap between inherited susceptibility and acquired triggers (1, 24).

Accordingly, even in apparently secondary HLH, broader genetic or metabolic workup should be considered in infants, atypical presentations, recurrent disease, or cases associated with unusual severity. At the same time, incomplete genetic testing remains an important limitation. In patients who were not genetically tested, classification as secondary HLH was supported by the presence of an identifiable associated trigger, absence of family history suggestive of familial HLH-like presentations, and lack of recurrence during follow-up after treatment of the precipitating condition. Nevertheless, occult inherited susceptibility or immune dysregulation cannot be completely excluded, and our classification should therefore be interpreted as clinically based rather than as definitive exclusion of all genetic predisposition.

This study has several limitations. It is a retrospective single-center study with a small sample size and marked etiologic heterogeneity, which restricts statistical power and precludes robust subgroup comparisons or identification of independent prognostic factors. Although all patients fulfilled at least five HLH-2004 criteria based on available data, diagnostic evaluation was not uniform across the study period, as NK-cell activity and sCD25 assays were unavailable at our center and genetic testing was performed in only a subset of patients. Therefore, although cases were classified as secondary HLH based on the presence of an associated trigger, compatible clinicopathologic findings, absence of suggestive family history, and lack of recurrence during follow-up in untested patients, occult inherited predisposition cannot be fully excluded. In addition, treatment was not protocolized and varied according to etiology, disease severity, and clinician judgment, which limits interpretation of regimen-specific outcomes, an additional limitation relates to disease classification, as the case with clinically supported lysinuric protein intolerance may reflect HLH associated with inherited metabolic predisposition rather than a purely acquired secondary form. Finally, although no HLH reactivation or relapse was documented among survivors during follow-up, late complications were not systematically captured.

Despite these limitations, our study provides useful real-world data from a tertiary pediatric center and supports several clinically important conclusions. Secondary HLH in children is most often triggered by infection and may have an excellent prognosis when recognized early and treated promptly. In contrast, HLH associated with metabolic disease, LCH, or other high-risk underlying conditions appear more refractory and may require more aggressive or disease-specific strategies. Overall, our findings underscore the importance of early recognition, rapid etiologic evaluation, tailored therapy, and selective genetic/metabolic investigation in the management of pediatric secondary HLH.

## Data Availability

The datasets presented in this article are not readily available because the primary data underlying this study are not publicly available in order to protect patient privacy and confidentiality. De-identified data may be made available by the corresponding author upon reasonable request, subject to approval by the King Hussein Cancer Center Institutional Review Board (KHCC-IRB). Requests to access the datasets should be directed to Mayada Abu Shanap, mayadaabushanap@gmail.com.
